# Professional Quality of Life and Occupational Stress Among Nurses in Pediatric Palliative and Related Pediatric Care Settings: A Cross-Sectional Study in Croatia

**DOI:** 10.3390/healthcare14172702

**Published:** 2026-08-24

**Authors:** Mia Rebernik, Krešimir Dolić, Matea Dolić

**Affiliations:** 1 Faculty of Health Sciences, University of Split, 21000 Split, Croatia; mia.rebernik1@gmail.com (M.R.); kdolic79@gmail.com (K.D.); 2 School of Medicine, University of Split, 21000 Split, Croatia; 3 Department of Radiology, University Hospital of Split, 21000 Split, Croatia

**Keywords:** burnout, nurses, pediatric palliative care, professional quality of life, secondary traumatic stress, occupational stress

## Abstract

**Highlights:**

**What are the main findings?**

- Participants reported high levels of compassion satisfaction, while burnout and secondary traumatic stress were present at low-to-moderate levels.
- Better organizational conditions, emotional support from colleagues, and access to professional education were associated with more favorable indicators of professional quality of life.

**What are the implications of the main findings?**

- Strengthening organizational and peer support structures may help promote more favorable professional quality-of-life outcomes among nurses working in emotionally demanding pediatric settings.
- Continuous education and targeted professional development programs should be integrated into pediatric palliative care services to support nurses’ well-being and sustain high-quality patient care.

**Abstract:**

**Background/Objectives:** Pediatric palliative care is a highly demanding field requiring nurses to maintain professional competence and emotional resilience. Continuous exposure to suffering, dying, and death may increase the risk of psychological distress among healthcare workers. The objective of this study was to examine compassion satisfaction, burnout, and secondary traumatic stress among nurses working in pediatric palliative and related pediatric care settings in Split-Dalmatia County, Croatia, and to explore organizational and emotional factors associated with their professional quality of life. **Methods:** A descriptive cross-sectional study was conducted involving 36 female nurses employed in a pediatric hospice, pediatric hemato-oncology department, and neonatology department. Data were collected using the Professional Quality of Life (ProQOL) questionnaire and a custom questionnaire assessing organizational, educational, and emotional challenges. **Results:** Participants reported high levels of compassion satisfaction (M = 4.55, SD = 0.45), moderate levels of secondary traumatic stress (M = 3.02, SD = 0.68), and relatively low levels of burnout (M = 2.34, SD = 0.65). A significant negative correlation was found between compassion satisfaction and burnout (ρ = −0.542; *p* < 0.001). Favorable organizational conditions, including sufficient time for rest (ρ = −0.567; *p* < 0.001) and emotional support from colleagues (ρ = −0.414; *p* = 0.012), were significantly associated with lower burnout levels. Although 63.9% of nurses lacked formal psychological support in the workplace, communication with families of terminally ill children remained a major emotional burden (M = 4.25, SD = 1.13) and was positively associated with secondary traumatic stress (ρ = 0.460; *p* = 0.005). **Conclusions:** Despite the substantial emotional demands of pediatric palliative and terminal care, nurses reported high compassion satisfaction alongside moderate secondary traumatic stress and relatively low burnout. The findings suggest that organizational conditions, collegial support, and access to formal psychological support may represent relevant targets for strategies aimed at sustaining nurses’ professional quality of life.

## 1. Introduction

Pediatric palliative care (PPC) represents a proactive, holistic, and multidisciplinary approach to relieving suffering in children facing life-threatening and life-limiting conditions [1]. The global need for PPC is increasing out of the continuous advancement in medical sciences, which prolongs the lives of children with complex chronic conditions, necessitating long-term health support [2].

Nurses form the backbone of PPC, spending the most direct time with terminally ill children and their families. Their roles extend beyond physical care to encompass extensive emotional, psychological, and spiritual support. Consequently, the pediatric palliative and hemato-oncology environments are emotionally fraught. Continuous exposure to children’s suffering, parental grief, and frequent patient deaths places nurses at a significantly heightened risk for psychological strain [3,4].

Occupational stress in this demographic frequently manifests as fatigue, workplace burnout, and secondary traumatic stress (STS). Burnout is an occupational syndrome resulting from chronic workplace stress that has not been successfully managed, characterized by emotional exhaustion, depersonalization, and a sense of reduced professional efficacy [5]. Over time, burnout among nurses directly compromises the quality of delivered care, patient safety, and the nurses’ own mental well-being [6]. Furthermore, ethical dilemmas—such as navigating treatment limitation decisions and aligning clinical guidelines with the holistic needs of the child—frequently lead to moral distress, further exacerbating the risk of burnout [7].

Despite the well-documented risks, factors such as compassion satisfaction (CS)—the pleasure derived from doing one’s work well and helping others—can serve as a protective buffer against burnout and STS [8]. The Job Demands–Resources (JD-R) model provides a useful conceptual framework for understanding occupational well-being by distinguishing between job demands, which may contribute to strain and exhaustion, and job resources, which may support motivation, resilience, and well-being. In the context of pediatric palliative care, emotional demands, exposure to suffering and death, complex communication with families, and workload may represent relevant job demands, whereas collegial support, opportunities for rest, professional education, and organizational support may function as job resources. In the present study, the JD-R model was used as a theoretical framework to support interpretation of the observed associations rather than to test causal relationships [9]. In Croatia, the systemic development of palliative care, particularly pediatric, is still in transitional phases, often facing organizational challenges like staff shortages, inadequate geographic coverage, and a lack of continuous specialized educational frameworks [10,11]. This study addresses a critical knowledge gap regarding the specific occupational stressors faced by PPC nurses in Croatia. A particular feature of pediatric palliative care development in Croatia is the leading role of Split-Dalmatia County in establishing specialized services for children with life-limiting and life-threatening conditions. In 2024, the first specialized mobile pediatric palliative care team in the country was established within the Split-Dalmatia County Health Center [11]. Furthermore, in 2025, a dedicated pediatric palliative care inpatient unit was opened within Hospice Matošić in Split, representing a major milestone in the development of pediatric palliative care services in Croatia [12]. These developments position Split-Dalmatia County as a pioneering regional model for the organization of pediatric palliative care and highlight the need to strengthen pediatric palliative care networks and workforce capacities across Croatia [13]. The primary aim of this study was to analyze the psychological, emotional, educational, and organizational challenges encountered by nurses in pediatric palliative and terminal care within Split-Dalmatia County. Specifically, we sought to: (1) assess levels of burnout, STS, and CS; (2) determine organizational and educational barriers; and (3) examine associations between professional quality of life and selected organizational, educational, and emotional workplace variables.

## 2. Materials and Methods

### 2.1. Study Design

This study used a cross-sectional design based on an anonymous online survey. The study explored professional quality of life, occupational stressors, and organizational, educational, and emotional challenges experienced by nurses involved in pediatric palliative care and related pediatric care settings.

### 2.2. Participants and Recruitment

A cross-sectional descriptive study was conducted in April 2026 among nurses employed in pediatric palliative and terminal care settings in Split-Dalmatia County, Croatia. Participants were recruited from a pediatric hospice of the Split-Dalmatia County Health Center, and the pediatric hemato-oncology department and neonatal intensive care units of the University Hospital of Split, all of which provide care for children with complex, life-limiting, or palliative care needs.

Eligible participants were nurses: (1) from a pediatric hospice, a pediatric hemato-oncology department, or a neonatal intensive care unit that provides care for children with complex, life-limiting, or palliative care needs, (2) had at least six months of work experience in their current position, (3) were employed within Split-Dalmatia County, and (4) voluntarily agreed to participate in the study.

A total of 53 nurses met the inclusion criteria and were invited to participate. The survey was distributed electronically via institutional e-mail and internal communication channels. Participants received an initial invitation followed by two reminder messages sent at one-week intervals. Of the 53 eligible nurses, 36 completed the questionnaire, resulting in a response rate of 67.9%.

Although the sample size was relatively small, it represented a substantial proportion of the available workforce involved in pediatric palliative and terminal care within the study region given the highly specialized nature of pediatric palliative care and the limited number of professionals working in this field. Although the sample represented a substantial proportion of the eligible workforce in the study region, caution is warranted when interpreting the findings because of the small sample size and possible non-response bias.

Before accessing the questionnaire, all participants received information regarding the purpose of the study, the voluntary nature of participation, data confidentiality, and anonymity. Completion of the questionnaire was considered as providing informed consent to participate in the study.

### 2.3. Instrument

Data were collected using an anonymous online questionnaire created in Google Forms. The survey consisted of three sections.

The first section collected sociodemographic and professional data, including age, marital status, parenthood, educational level, current work setting, years of work experience, employment status, and work schedule.

The second section included the Professional Quality of Life Scale, Version 5 (ProQOL v5), a widely used 30-item self-report instrument designed to assess three dimensions of professional quality of life: Compassion Satisfaction (CS), Burnout (BO), and Secondary Traumatic Stress (STS) [14]. Each subscale contains 10 items rated on a 5-point Likert scale ranging from 1 (“never”) to 5 (“very often”). In accordance with the ProQOL v5 scoring instructions, Burnout items 1, 4, 15, 17, and 29 were reverse-scored prior to calculation. For each subscale, summed scores were used for categorical interpretation according to Stamm, with scores of 22 or less indicating low levels, 23–41 moderate levels, and 42 or more high levels. Mean item scores are presented in the manuscript for descriptive clarity, while categorical classifications were based on summed subscale scores [14]. The Professional Quality of Life Scale (ProQOL-5) was used to assess three dimensions of professional quality of life: Compassion Satisfaction, Burnout, and Secondary Traumatic Stress. Previous research involving Croatian nurses demonstrated good internal consistency of the ProQOL, with Cronbach’s α coefficients of 0.88 for Compassion Satisfaction, 0.79 for Burnout, and 0.78 for Secondary Traumatic Stress [15]. More recently, a study conducted among nurses and nursing technicians in Croatia reported Cronbach’s α coefficients of 0.92, 0.78, and 0.87 for Compassion Satisfaction, Burnout, and Secondary Traumatic Stress, respectively [16]. In the present study, the ProQOL demonstrated similarly good internal consistency, with Cronbach’s α coefficients of 0.835 for Compassion Satisfaction, 0.836 for Burnout, and 0.778 for Secondary Traumatic Stress. Overall, these findings support the reliability of the ProQOL for assessing professional quality of life among Croatian healthcare professionals [15,16].

The third section consisted of a study-specific workplace questionnaire developed for the purposes of this research. It included items addressing organizational, educational, ethical, and emotional aspects of work with terminally ill children. Educational aspects were assessed using two separate single-item indicators: (1) perceived availability of specialized education, referring to participants’ perception of the availability of specialized pediatric palliative care (PPC) training opportunities, and (2) perceived sufficient education, referring to participants’ self-assessment of whether they had adequate knowledge and skills to provide PPC. These two items were analyzed as separate variables.

Other workplace variables included perceived time for rest, emotional support from colleagues and family, communication with patients’ families as an emotional burden, and thoughts about changing jobs. Items were rated on a 5-point Likert scale. Several key workplace variables were measured using single-item indicators; therefore, findings based on this section should be interpreted as exploratory. The full item list is provided in the Supplementary Materials.

### 2.4. Data Collection

Data were collected through an online survey over a three-week period. The survey link was distributed via email and internal communication channels by palliative care coordinators in the participating institutions. After the initial invitation, two reminder messages were sent at one-week intervals.

### 2.5. Statistical Analysis

Data were analyzed using IBM SPSS Statistics (version: 26.0). Categorical variables are presented as frequencies and percentages, while continuous variables are presented as means and standard deviations. The normality of continuous variables was assessed using the Shapiro–Wilk test, and the homogeneity of variances was assessed using Levene’s test.

Differences between nurses working in pediatric hospice, pediatric hemato-oncology, and neonatology were examined using one-way analysis of variance (ANOVA) when the assumptions of normality and homogeneity of variances were met. The Kruskal–Wallis test was additionally performed as a sensitivity analysis to verify the robustness of the findings. For comparisons between two independent groups, the Mann–Whitney U test was used, where appropriate. Associations between continuous or ordinal variables were assessed using Spearman’s rank-order correlation coefficient, while associations between categorical variables were examined using Pearson’s chi-square test.

All statistical tests were two-sided, and statistical significance was set at *p* < 0.05. Because the study was exploratory and based on a relatively small sample, the primary analytic approach focused on unadjusted bivariate associations. No formal correction for multiple testing was applied; therefore, the findings should be interpreted cautiously. Bias-corrected and accelerated (BCa) bootstrap confidence intervals based on 5000 resamples were calculated for Spearman correlation coefficients.

All submitted questionnaires were screened for completeness before analysis. The online questionnaire required responses to all questionnaire items before submission; therefore, there were no missing item-level data.

### 2.6. Ethical Considerations

The survey was anonymous and voluntary. Participants were informed about the study purpose and gave informed consent before participation. No personal identifying information was collected. The study obtained approval from the Ethics Committee of the Split-Dalmatia County Health Center (Class: 007-04/26-06/004; Reg. No.: 2181-149-016-26-002) and the University Hospital of Split (Class: 520-03/26-01/68, Reg. No.: 2181-147/01-06/LJ.U.-26-02). Electronic informed consent was obtained from all participants prior to accessing the questionnaire.

## 3. Results

### 3.1. Sociodemographic and Professional Characteristics

The study included 36 nurses, all of whom were female. The largest proportion of participants were aged 25–35 years (47.2%), married (66.7%), and had children (69.4%). Nearly half of the participants had completed secondary nursing education (47.2%), while 52.8% reported more than 10 years of professional experience in healthcare. Most participants worked in neonatology (58.3%), performed shift work (91.7%), and were employed full-time (100.0%). Detailed sociodemographic and professional characteristics are presented in Table 1.

### 3.2. Professional Quality of Life

ProQOL scores were further categorized according to the standard classification proposed by Stamm (2010) [14], using the established cut-off values for low, moderate, and high levels of Compassion Satisfaction (CS), Burnout (BO), and Secondary Traumatic Stress (STS).

The categorization of ProQOL scores revealed a highly favorable professional quality of life profile among participants. More than three-quarters of nurses (77.8%) reported high levels of Compassion Satisfaction, while the remaining 22.2% fell within the moderate category. No participant demonstrated low Compassion Satisfaction.

Regarding Burnout, participants were equally distributed between the low (50.0%) and moderate (50.0%) categories, with no respondents reaching the high burnout category. Similarly, Secondary Traumatic Stress was predominantly observed at a moderate level (86.1%), whereas 13.9% of participants reported low levels of secondary traumatic stress. No participant scored within the high STS category.

These findings are consistent with the mean ProQOL scores reported previously. The high mean Compassion Satisfaction score (M = 4.55) corresponds to the predominance of participants in the high CS category. The mean Burnout score (M = 2.34) reflects the distribution of respondents between the low and moderate burnout categories, while the mean Secondary Traumatic Stress score (M = 3.02) is consistent with the predominance of moderate STS levels. Overall, the categorical analysis confirms that nurses working in pediatric palliative and terminal care experience high professional fulfillment alongside relatively low levels of occupational distress (Table 2).

### 3.3. Differences in ProQOL Subscales Across Work Settings

A one-way analysis of variance (ANOVA) revealed no statistically significant differences among participants employed in different work settings regarding compassion satisfaction (F(2, 33) = 0.255, *p* = 0.776, η^2^ = 0.015), burnout (F(2, 33) = 0.754, *p* = 0.478, η^2^ = 0.044), or secondary traumatic stress (F(2, 33) = 0.371, *p* = 0.693, η^2^ = 0.022).

The assumption of homogeneity of variances was met for all analyzed variables, as confirmed by Levene’s test for compassion satisfaction (*p* = 0.851), burnout (*p* = 0.795), and secondary traumatic stress (*p* = 0.654). Furthermore, the application of the nonparametric Kruskal–Wallis test yielded consistent results, indicating no statistically significant differences between the groups for any of the observed variables (Table 3).

### 3.4. Correlations Among ProQOL Subscales

Spearman’s rank-order correlation showed a statistically significant moderate negative correlation between compassion satisfaction and burnout (ρ = −0.542, *p* < 0.001), and a statistically significant moderate positive correlation between burnout and secondary traumatic stress (*p* = 0.540, *p* < 0.001). No statistically significant correlation was observed between compassion satisfaction and secondary traumatic stress (*p* = −0.152, *p* = 0.377) (Table 4).

### 3.5. Associations Between ProQOL and Workplace Variables

Spearman’s rank-order correlation was used to examine associations between ProQOL subscales and selected workplace variables. Burnout was negatively associated with perceived time for rest (ρ = −0.567, *p* < 0.001), perceived availability of specialized education (ρ = −0.402, *p* = 0.015), and emotional support from colleagues (ρ = −0.414, *p* = 0.012). Burnout was positively associated with communication with families as an emotional burden (ρ = 0.380, *p* = 0.022) and thoughts about changing jobs (ρ = 0.665, *p* < 0.001).

Compassion satisfaction was positively associated with the perceived availability of specialized education (ρ = 0.364, *p* = 0.029). Secondary traumatic stress was positively associated with communication with families as an emotional burden (ρ = 0.460, *p* = 0.005). In addition, perceived time for rest was positively associated with emotional support from colleagues (ρ = 0.591, *p* < 0.001) and negatively associated with thoughts about changing jobs (ρ = −0.692, *p* < 0.001). Emotional support from colleagues was also negatively associated with thoughts about changing jobs (ρ = −0.409, *p* = 0.013).

All 95% BCa bootstrap confidence intervals excluded zero for the statistically significant associations presented in Table 5.

### 3.6. Organizational, Educational, and Emotional Challenges

Participants reported an acute need for continuous professional development (M = 4.31, SD = 0.86) and evaluated the availability of specialized PPC training as insufficient (M = 2.56, SD = 1.11). In addition to assessing the availability of specialized education, participants also evaluated whether they considered themselves sufficiently educated to provide pediatric palliative care. More positive perceptions of being sufficiently educated were significantly associated with higher compassion satisfaction (ρ = 0.593, *p* < 0.001).

Emotionally, communicating with the families of terminally ill children constituted a substantial emotional burden (M = 4.25, SD = 1.13). Crucially, 63.9% of the surveyed nurses reported lacking access to professional psychological support at their workplace. Consequently, participants heavily relied on emotional support from colleagues (M = 3.89, SD = 1.14) and family (M = 3.83, SD = 1.36). Higher emotional support from colleagues was significantly linked to lower burnout (ρ = −0.414; *p* = 0.012) and fewer thoughts about leaving their current job (ρ = −0.409; *p* = 0.013).

## 4. Discussion

This study examined professional quality of life and selected workplace correlates among nurses working in pediatric hospice/palliative care, pediatric hemato-oncology, and neonatology in Split-Dalmatia County, Croatia. The findings indicate a mixed but clinically relevant pattern: participants reported high compassion satisfaction, moderate secondary traumatic stress, and low-to-moderate burnout. At the bivariate level, higher compassion satisfaction was associated with lower burnout, whereas lower burnout was associated with more favorable perceptions of time for rest, emotional support from colleagues, and access to specialized education.

These findings may be interpreted within the Job Demands-Resources (JD-R) framework, which proposes that employee well-being reflects the balance between workplace demands and available personal and organizational resources [9]. In the present study, compassion satisfaction may be understood as a potentially relevant personal resource, while collegial support and opportunities for rest may be understood as workplace resources associated with more favorable professional quality of life. This interpretation is also consistent with evidence that organizational and psychosocial resources may function as protective factors against burnout among healthcare workers [17,18,19,20,21,22]. However, these interpretations should remain theoretical rather than causal as the present design does not allow for conclusions about directionality or mechanism.

The inverse association between compassion satisfaction and burnout is consistent with previous literature suggesting that professional fulfillment and meaning in caregiving are linked to more favorable well-being outcomes among nurses. Recent work using the ProQOL framework likewise supports the relevance of compassion satisfaction, burnout, and secondary traumatic stress as interrelated dimensions of occupational well-being in demanding clinical settings [8,14,15,16]. Similarly, studies of healthcare professionals have identified burnout as a multidimensional occupational phenomenon influenced by workload, organizational conditions, interpersonal relationships, and available protective resources [17,18,19,20,21,22]. In palliative care, factors related to social and psychological support may be particularly important given the sustained emotional demands of caring for seriously ill and dying patients and their families [6,23].

At the same time, the associations observed in this study should be interpreted cautiously. Because all variables were measured concurrently and through self-report, the correlations may partly reflect overlapping perceptions of workplace functioning, distress, and coping, rather than independent relationships between clearly separable constructs. For example, the association between burnout and thoughts about changing jobs may reflect shared distress-related response tendencies, while the associations of burnout with rest and collegial support may also be shaped by workload, departmental culture, or unmeasured organizational conditions. This interpretation is consistent with the broader literature indicating that burnout among healthcare professionals is influenced by multiple interacting individual, occupational, and organizational factors rather than by a single determinant [17,18,19,20,21,22].

The finding that communication with families of terminally ill children was associated with higher secondary traumatic stress is clinically plausible and aligns with literature describing pediatric and palliative care as intensely emotional practice environments [2,4,7]. Difficult conversations, repeated exposure to grief, and ongoing emotional engagement with families may place a substantial burden on nurses even when overall burnout levels remain relatively low. Emotional labor has been specifically identified as an important component of pediatric palliative care practice, reflecting the need to simultaneously manage one’s own emotional responses and provide compassionate support to children and families [4]. Furthermore, staff working with children and adolescents who present complex or challenging situations may experience substantial emotional and relational demands across different pediatric care environments [24]. In this context, the strong expressed need for continuing professional development may reflect not only educational needs, but also the emotional and ethical complexity of this area of practice [3,4,7].

A particularly important contextual finding was that many participants reported lacking formal psychological support in the workplace. This is relevant because respondents appeared to rely heavily on informal support from colleagues and family. Previous research in palliative care has emphasized unmet social, psychological, and spiritual needs and the importance of adequate support structures for professionals working in this field [23]. Similarly, the literature on pediatric palliative care highlights the need for appropriate preparation, education, communication skills, and organizational support for healthcare professionals [2,3,7]. The importance of supportive work environments is also consistent with the JD-R framework, in which social and organizational resources may help buffer the effects of demanding work conditions [9].

The need for structured approaches to staff well-being is further supported by emerging intervention-oriented evidence. Marinetto et al. reported the potential of virtual reality as a tool to promote healthcare provider well-being in pediatric palliative care [25], suggesting that innovative approaches to staff support may have a role alongside conventional organizational and psychosocial interventions. Although such interventions should not be interpreted as substitutes for adequate staffing, rest, supervision, or psychological support, they illustrate the growing recognition that staff well-being requires deliberate organizational attention.

The absence of statistically significant differences across the surveyed work settings should also be interpreted with caution. Although no significant between-group differences were detected, the subgroup sizes were small, particularly in the pediatric hospice/palliative care and pediatric hemato-oncology groups, and the study was not powered to reliably detect modest setting-related differences. Accordingly, the present findings should not be interpreted as evidence that work setting is unrelated to professional quality of life, but rather that such differences could not be meaningfully evaluated in this sample.

Overall, this study contributes preliminary evidence from a Croatian setting in which pediatric palliative care remains organizationally developing and unevenly supported [10,11,12,13]. Its primary contribution is not in identifying determinants, but in showing that professional quality of life among nurses in these settings is associated with perceived workplace and support conditions, and that formal psychological support structures may be insufficient in relation to the emotional demands of care. This interpretation is particularly relevant in the Croatian context, where the development of pediatric palliative services has been accompanied by an increasing need for appropriately trained professionals and sustainable models of support [10,11,12,13]. The findings also complement previous Croatian evidence concerning unmet social, psychological, and spiritual needs in palliative care, highlighting that the needs of professionals and patients should be considered within the broader development of palliative care systems [23].

Non-response bias cannot be excluded, as 17 of the 53 eligible nurses did not participate and no data were available to compare responders and non-responders.

### 4.1. Implications for Practice

The findings of this study highlight the need for systematic development of support strategies for nurses involved in pediatric palliative care. Particular attention should be directed toward ensuring continuous professional education, providing access to emotional support, and developing organizational strategies aimed at preserving professional quality of life [3,4,6,9,17].

The implementation of programs focused on burnout prevention, strengthening emotional resilience, and promoting team support could contribute to reducing the negative consequences of emotionally demanding work and enhancing nurses’ compassion satisfaction [18,19,20,22]. Interventions should ideally address both the individual and organizational levels, consistent with the JD-R framework, rather than placing responsibility for coping exclusively on individual healthcare professionals [9].

Improving working conditions and developing specialized educational programs in pediatric palliative care could have a positive impact not only on healthcare professionals’ well-being but also on the quality of care provided to children and their families [1,2,3,4,7]. Structured opportunities for professional development, team-based support, psychological assistance, and debriefing following particularly difficult clinical situations may therefore represent important components of a comprehensive staff-support strategy [3,4,7,23]. Innovative approaches, including technology-assisted well-being interventions, can also be considered as complementary strategies in pediatric palliative care settings [25].

### 4.2. Strengths and Limitations

Several limitations should be considered when interpreting these findings. First, the sample was small and drawn from a single Croatian county, which limits the statistical power and reduces the generalizability of the results. In particular, the subgroup analyses by work setting were based on small numbers and therefore had limited capacity to detect between-setting differences. Second, the study used a cross-sectional design so temporal ordering cannot be established. The observed relationships should therefore be interpreted strictly as associations and not as evidence of causal or directional effects. Third, all predictors and outcomes were measured by self-report at a single timepoint, which raises the possibility of common-method variance and shared response-style bias. This may have inflated some of the observed correlations, especially those involving single-item workplace indicators and distress-related perceptions. Fourth, the workplace questionnaire used in addition to the ProQOL instrument was developed for the purpose of this study and included several single-item indicators. Although these items were clinically relevant, their psychometric properties were not formally established, so their findings should be regarded as exploratory. Fifth, the possibility of non-response bias cannot be excluded. Seventeen of the 53 eligible nurses did not participate, and no data were available to compare responders and non-responders. If nurses experiencing higher burden, disengagement, or dissatisfaction were less likely to respond, the present findings may overestimate favorable professional quality-of-life patterns.

Finally, because multiple statistical tests were conducted in an exploratory dataset without formal multiplicity correction, the findings should be interpreted cautiously and confirmed in larger, preferably multicenter studies with adjusted analyses.

## 5. Conclusions

Among nurses working in pediatric hospice/palliative care and related pediatric hospital settings in Split-Dalmatia County, compassion satisfaction was high, secondary traumatic stress was moderate, and burnout was low-to-moderate. Better professional quality of life was associated with more favorable perceived workplace conditions, including greater time for rest, stronger collegial support, and more positive perceptions of specialized education. These findings should be interpreted as exploratory and associative rather than causal. The findings highlight the potential relevance of organizational and psychological support structures in emotionally demanding pediatric care environments, including opportunities for rest, peer support, debriefing, and targeted education. Larger studies are needed to clarify whether these associations persist after adjustment for work setting and professional characteristics and to evaluate the potential effectiveness of specific interventions for supporting nurses in pediatric palliative and related care settings.

## Figures and Tables

**Table 1:** Sociodemographic and professional characteristics of the participants (*N* = 36).

Characteristic	Category	*n* (%)
**Sex**	Female	36 (100.0)
	Male	0 (0.0)
**Age**	25–35 years	17 (47.2)
	36–45 years	8 (22.2)
	46–60 years	11 (30.6)
**Marital status**	Single	4 (11.1)
	Married	24 (66.7)
	In a relationship	7 (19.4)
	Divorced	1 (2.8)
	Widowed	0 (0.0)
**Children**	Yes	25 (69.4)
	No	11 (30.6)
**Highest educational level**	Secondary nursing school	17 (47.2)
	Bachelor’s degree	11 (30.6)
	Graduate nursing degree	5 (13.9)
	Master’s degree	3 (8.3)
	Doctoral degree	0 (0.0)
**Years of experience in healthcare**	0–10 years	17 (47.2)
	≥11 years	19 (52.8)
**Years of experience in pediatric/palliative care**	0–5 years	16 (44.4)
	6–15 years	6 (16.7)
	16–25 years	6 (16.7)
	26–38 years	8 (22.2)
**Current work setting**	Children’s hospice	7 (19.4)
	Pediatric hemato-oncology	8 (22.2)
	Neonatology	21 (58.3)
**Shift work**	Yes	33 (91.7)
	No	3 (8.3)
**Employment status**	Full-time	36 (100.0)
	Part-time	0 (0.0)
**Average weekly working hours**	30–39 h	5 (13.9)
	40–44 h	13 (36.1)
	45–50 h	18 (50.0)
**Years working with seriously ill or terminally ill patients**	≤3 years	10 (27.8)
	4–10 years	9 (25.0)
	>10 years	17 (47.2)

**Table 2:** ProQOL categorization (low/moderate/high).

Subscale	Low	Moderate	High
Compassion Satisfaction	0%	22.2%	77.8%
Burnout	50.0%	50.0%	0%
Secondary Traumatic Stress	13.9%	86.1%	0%

Note: Categories were based on summed ProQOL subscale scores according to Stamm (2010) [14]: ≤22 = low, 23–41 = moderate, and ≥42 = high.

**Table 3:** Differences in ProQOL subscales across work settings.

Outcome	F	df	*p*-Value	Eta Squared ($\eta^{2}$)	Levene’s *p*-Value
Compassion satisfaction	0.255	2, 33	0.776	0.015	0.851
Burnout	0.754	2, 33	0.478	0.044	0.795
Secondary traumatic stress	0.371	2, 33	0.693	0.022	0.654

Note: Group comparisons were performed across pediatric hospice/palliative care, pediatric hemato-oncology, and neonatology settings. The Kruskal–Wallis test yielded the same pattern of non-significant results.

**Table 4:** Spearman correlations among ProQOL subscales.

Variable Pair	Spearman’s rho	*p*-Value
Compassion satisfaction—Burnout	−0.542	<0.001
Compassion satisfaction—Secondary traumatic stress	−0.152	0.377
Burnout—Secondary traumatic stress	0.540	<0.001

**Table 5:** Spearman correlations between ProQOL subscales and selected workplace variables (*n* = 36).

Variables	Spearman’s rho	95% BCa Bootstrap CI	*p*-Value
Compassion satisfaction—Burnout	−0.542	[−0.769, −0.222]	<0.001
Compassion satisfaction—Secondary traumatic stress	−0.152	[−0.490, 0.210]	0.377
Burnout—Secondary traumatic stress	0.540	[0.248, 0.746]	<0.001
Burnout—Time for rest	−0.567	[−0.774, −0.266]	<0.001
Burnout—Availability of specialized education	−0.402	[−0.658, −0.045]	0.015
Burnout—Emotional support from colleagues	−0.414	[−0.678, −0.082]	0.012
Burnout—Communication with families as emotional burden	0.380	[0.060, 0.647]	0.022
Burnout—Thoughts about changing jobs	0.665	[0.397, 0.834]	<0.001
Compassion satisfaction—Availability of specialized education	0.364	[0.032, 0.656]	0.029
Secondary traumatic stress—Communication with families as emotional burden	0.460	[0.174, 0.691]	0.005
Time for rest—Emotional support from colleagues	0.591	[0.320, 0.794]	<0.001
Time for rest—Thoughts about changing jobs	−0.692	[−0.813, −0.504]	<0.001
Emotional support from colleagues—Thoughts about changing jobs	−0.409	[−0.680, −0.082]	0.013

BCa, bias-corrected and accelerated; CI, confidence interval; ProQOL, Professional Quality of Life. Note: Availability of specialized education refers to the perceived availability of specialized PPC training opportunities. Perceived sufficient education refers to participants’ self-assessment of having adequate knowledge and skills for pediatric palliative care and is reported separately in Section 3.6.

## Data Availability

The data presented in this study are available from the corresponding author upon reasonable request. The data are not publicly available due to privacy and ethical restrictions related to the protection of participants’ confidentiality.

## Supplementary Materials

The following supporting information can be downloaded at: https://www.mdpi.com/article/10.3390/healthcare14172702/s1. Supplementary Table S1. Full pairwise Spearman correlation matrix (*N* = 36).
