# Supplementary material for: Professional Quality of Life and Occupational Stress Among Nurses in Pediatric Palliative and Related Pediatric Care Settings: A Cross-Sectional Study in Croatia
Source: Healthcare (Basel). 2026 Aug 24;14(17):2702. doi: 10.3390/healthcare14172702 (PMC13565740; doi:10.3390/healthcare14172702)
Supplement: Supplementary file 1 [file healthcare-14-02702-s001.zip › healthcare-4504978-supplementary.pdf]

**Supplementary Table S1. Full pairwise Spearman correlation matrix (N = 36)**

| Variable | 1 | 2 | 3 | 4 | 5 | 6 | 7 | 8 | 9 | 10 | 11 | 12 | 13 | 14 | 15 | 16 |
|---|---|---|---|---|---|---|---|---|---|---|---|---|---|---|---|---|
| 1 | — | | | | | | | | | | | | | | | |
| 2 | -.329 | — | | | | | | | | | | | | | | |
| 3 | -.340* | .407* | — | | | | | | | | | | | | | |
| 4 | .369* | .205 | -.328 | — | | | | | | | | | | | | |
| 5 | -.133 | .186 | .279 | -.008 | — | | | | | | | | | | | |
| 6 | .040 | .036 | -.253 | .255 | .397* | — | | | | | | | | | | |
| 7 | -.074 | .093 | .223 | .168 | .431** | .148 | — | | | | | | | | | |
| 8 | -.005 | .074 | .207 | .095 | .462** | .306 | .318 | — | | | | | | | | |
| 9 | -.052 | .591** | .332* | .048 | .386* | .130 | .277 | .202 | — | | | | | | | |
| 10 | .111 | -.198 | -.232 | .340* | -.051 | .009 | .103 | .012 | -.304 | — | | | | | | |
| 11 | .235 | -.692** | -.240 | -.345* | -.259 | -.042 | -.121 | -.235 | -.409* | .249 | — | | | | | |
| 12 | .026 | -.053 | .006 | .017 | -.347* | -.186 | -.002 | -.124 | -.183 | .016 | -.159 | — | | | | |
| 13 | .047 | .315 | .364* | .109 | .593** | .072 | .587** | .435** | .315 | -.217 | -.324 | .027 | — | | | |
| 14 | .174 | -.567** | -.402* | -.048 | -.322 | -.075 | -.088 | -.405* | -.414* | .380* | .665** | -.100 | -.542** | — | | |
| 15 | .008 | -.180 | -.027 | .004 | -.064 | -.054 | .204 | -.080 | -.103 | .460** | .327 | -.072 | -.152 | .540** | — | |
| 16 | -.644** | .804** | .765** | -.157 | .242 | -.129 | .126 | .095 | .443** | -.226 | -.539** | -.012 | .248 | -.521** | -.084 | — |

**Variable names**

1. The geographical dispersion of the county (islands, rural areas) makes access to 24/7 care more difficult.
2. I have enough time for rest and recovery between shifts.
3. The availability of specialized education in pediatric palliative care is sufficient.
4. I feel the need for continuous professional development.
5. I consider myself sufficiently educated to work in pediatric palliative care.
6. I participate in decision-making regarding the organization of therapy.
7. I feel competent in assessing the best interests of the child.
8. I believe that parents have appropriate autonomy in care-related decisions.

9. I receive emotional support from colleagues in the workplace.
10. Communication with the family of a child in the terminal stage of illness represents a major emotional burden for me.
11. I have considered changing my workplace because of emotional burden.
12. My family and friends provide me with emotional support after a difficult shift.
13. ZS_mean
14. Burnout_mean
15. STstress_mean
16. Organization_mean

p < .05; ** p < .01 (two-tailed).

**Note:** The first three variables together form the construct represented by the composite variable Organization mean. The first variable, relating to the geographical dispersion of the county, was formulated and analyzed in this table in the negative direction; however, for the purpose of calculating the composite variable, it was reverse-coded to ensure alignment with the remaining two variables.
